# Clonality and Diversity in the Soft Rot *Dickeya solani* Phytopathogen

**DOI:** 10.3390/ijms242417553

**Published:** 2023-12-16

**Authors:** Frédérique Van Gijsegem, Perrine Portier, Géraldine Taghouti, Jacques Pédron

**Affiliations:** 1 Institute of Ecology and Environmental Sciences-Paris, Sorbonne Université, INRAE, 4 Place Jussieu, F-75252 Paris, France; jacques.pedron@upmc.fr; 2 Univ Angers, Institut Agro, INRAE, IRHS, SFR QUASAV, CIRM-CFBP, F-49000 Angers, France; perrine.portier@inrae.fr (P.P.); geraldine.taghouti@inrae.fr (G.T.)

**Keywords:** *Pectobacteriaceae*, comparative genomics, potato, soft rot, *Dickeya solani*

## Abstract

Bacterial diversity analyses often suffer from a bias due to sampling only from a limited number of hosts or narrow geographic locations. This was the case for the phytopathogenic species *Dickeya solani*, whose members were mainly isolated from a few hosts–potato and ornamentals–and from the same geographical area–Europe and Israel, which are connected by seed trade. Most *D. solani* members were clonal with the notable exception of the potato isolate RNS05.1.2A and two related strains that are clearly distinct from other *D. solani* genomes. To investigate if *D. solani* genomic diversity might be broadened by analysis of strains isolated from other environments, we analysed new strains isolated from ornamentals and from river water as well as strain CFBP 5647 isolated from tomato in the Caribbean island Guadeloupe. While water strains were clonal to RNS05.1.2A, the Caribbean tomato strain formed a third clade. The genomes of the three clades are highly syntenic; they shared almost 3900 protein families, and clade-specific genes were mainly included in genomic islands of extrachromosomal origin. Our study thus revealed both broader *D. solani* diversity with the characterisation of a third clade isolated in Latin America and a very high genomic conservation between clade members.

## 1. Introduction

Different forces including spreading via worldwide trade routes, dissemination to different hosts or survival in various environments drive structures of plant pathogen populations. This results in various degrees of genomic diversity from one bacterial species to another. The genus *Dickeya* is one example of such variations in diversity depending on the species [1,2].

*Dickeya* members are collectively responsible for soft rot, blackleg, stem rots, stunting, cankers and wilting on a large number of plant hosts, both monocots and dicots, including mostly vegetables and ornamentals but also trees [3]. Their most important virulence factor is the production and secretion of a battery of plant cell-wall-degrading enzymes that provoke the maceration of plant tissues, leading to cell lysis and release of nutrients. However, virulence also relies on several other factors that allow these bacteria to adapt to environmental changes encountered in planta and to face the stresses produced by plant defence responses [4].

The *Dickeya* genus consists of 13 species presenting differences in environmental habitats, plant host range and genomic diversity [1,3,5,6,7]. Among the species with a sufficient number of genomes available for diversity studies, several like *D. fanghzongdai*, *D. dadantii*, *D. chrysanthemi* and the *D. zeae* complex harbour a high genomic diversity with some strains being at the limit of species definition [1,2,8]. The previously described *D. zeae* species was even recently split into three species–*D. zeae*, *D. oryzae* and *D. parazeae* [2,6,7], but some strains of the two first species are still highly divergent from the type strains [2]. By contrast, *D. dianthicola* and *D. solani* members have been reported as genomically closely related [1,9,10,11,12,13,14]. Both species were responsible for important outbreaks in the same host potato, and consequently, most available genomes belong to isolates from this host plant. This introduced a bias due to the analysis mainly of strains isolated from only one host and only from Europe and the US. A recent study analysing *D. dianthicola* genomic diversity using a larger strain panel encompassing more strains isolated from potato over a wider time scale and more strains isolated from hosts other than potato revealed a higher diversity [15].

The host range of *D. solani* is quite narrow. This species was first described following an important outbreak ravaging potato in Europe in the 2000s [11]. Besides potato, it has been isolated only from two ornamentals, hyacinth and muscari [3]. Even if these strains have been isolated over a long time period [16], most of them are clonal, differing only from a few dozen to a few hundred SNPs [12,13,14,15,16,17]. The potato isolate RNS05.1.2A, however, is clearly distinct from other *D. solani* genomes, harbouring more than 30,000 SNPs with them [16]. Recently, two other strains isolated either from potato or from river water were shown to belong to this RNS05.1.2A clade [14]. A few genomes harbour medium diversity due to replacing horizontal gene transfers from *D. dianthicola* or RNS05.1.2A in the main core strain genome [16]. Concerning the accessory genomes, genomic variations in *D. solani* consist mainly in the presence of genes related to phages or even complete prophages [14,18]. Despite their very high genetic homogeneity, *D. solani* strains may vary considerably in their virulence levels (aggressiveness) as well as in their production of virulence factors and motility [18]. Analysis of *D. solani* biodiversity at the proteome level using matrix-assisted methods has unveiled intraspecies variations among 20 *D. solani* strains differing in their virulence-associated features in addition to the country of origin and year of isolation. These strains were grouped into four delineated clades and differed also in growth rate, plant tissue macerating potential and protease activities, in contrast to the high uniformity among the metabolic profiles, which diverged only in terms of gelatinase activity. However, a definitive link between pathogenicity and the recorded MALDI-TOF MS spectra could not be established [19].

In this work, we investigated if *D. solani* genomic diversity might be broadened through analysis of strains isolated from other environments: ornamentals, river water and a new host.

## 2. Results

### 2.1. Collection of New D. solani Strains Isolated in Different Environments

To broaden the sources of *D. solani* strains analysed for genomic diversity, we searched for strains isolated in environments other than potato (Table 1).

As the genomes of only two *D. solani* strains isolated from ornamentals have been analysed so far, we analysed two other strains isolated from hyacinth in the Netherlands: PO2019 and PO3796 (courtesy of Jan van der Wolf). We also took advantage of the two-year survey of soft rot *Pectobacteriaceae* we recently conducted in the Durance catchment, a river from the south of France [20] to chase *D. solani* isolates from river water. Four isolates were retrieved: FVG2-MFV017-A9 isolated from the Canal de Crillon, an irrigation canal fed by the river Durance, FVG9-S3-A17-E1 isolated from the Durance River itself and FVG13-S21A17-D9 and FVG14-S21A17-C8, both from a tributary of the Durance, the Grand Anguillon river (see Material and Methods for isolation procedure). New *D. solani* isolates genomically divergent from the type strain were also searched in the French Collection for Plant-associated Bacteria, CIRM-CFBP (https://cirm-cfbp.fr/; accesses on 12 December 2023), by using multilocus sequence analysis (MLSA) performed on the basis of *recA*, *leuS* and *dnaX* housekeeping genes. This allowed us to identify strain CFBP 5647 (isolated in Guadeloupe from tomato) that was mapped in a branch of the MLSA tree distinct both from the type strain and from RNS05.1.2A (Figure S1). The genomic characteristics of these seven strains are presented in Table 1. Furthermore, the complete sequence of CFBP 5647 was obtained using a hybrid assembly of Illumina short reads and ONT long reads. It consists of a single circular chromosome totalling 5,248,586 bp with a GC content of 56.2%. Annotation of this genome using the Rapid Annotation using Subsystems Technology (RAST) generated a total of 4978 predicted genes, including 4883 protein coding sequences, 74 tRNAs and 21 rRNAs organised in seven operons.

The accession numbers of the genome sequences presented here that were deposited at NCBI are presented in Table 1.

### 2.2. D. solani Genomic Diversity

#### 2.2.1. The Relatedness between *D. solani* Members

To analyse the *D. solani* diversity, we compared our seven new genomes with the genomes of the three *D. solani* strains of the minor subgroup exemplified by strain RNS05.1.2A [14,16] and seven members of the previously defined main subgroup here defined as the “core” clade. Out of the 40 “core” genomes present in the NCBI database, we chose the genomes of the type strain IPO2222, the two strains isolated from ornamentals PPO9019 and PPO9134, the strain RNS07.7.3B that harbours horizontal genetic transfers from strain RNS05.1.2A [16], the well-studied strains RNS08.23.3.1.A and Ds0432-1 [16,21,22,23,24] and the strain D12 from Russia, far from the Western European seed trade network.

**Figure 1:**
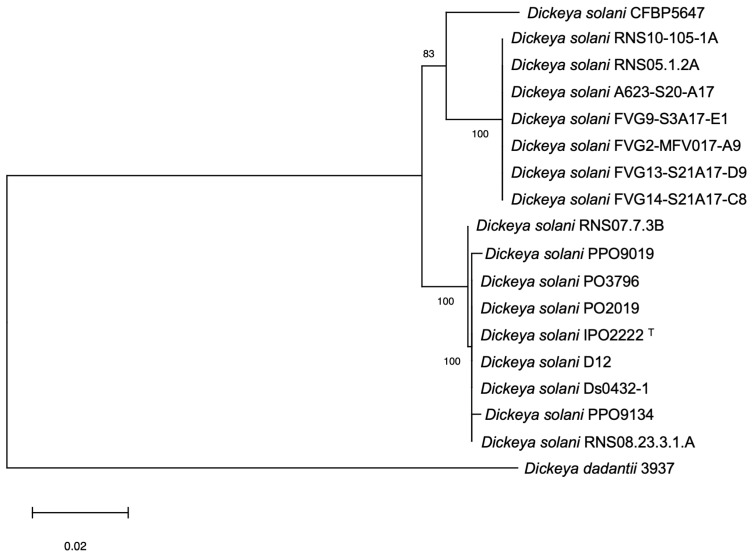
Whole genome MLSA phylogenic tree. Phylogenic tree built up from the concatenated sequences of 3403 homologous protein sequences (197,918 variable sites). One hundred bootstrap replicates were performed to assess the statistical support of each node.

A whole genome multilocus sequence analysis (MLSA) phylogenomic tree built up from concatenated sequences of 3403 core proteins (Figure 1) showed that all strains isolated from French watersheds belong to the minor clade related to RNS05.1.2A, while the two additional strains isolated from ornamentals belong to the core clade. Strains within each of these clades appeared highly related. The Caribbean strain CFBP 5647 isolated from tomato forms a third “clade”.

Calculation of average nucleotide identity (ANI) values between all genomes of our strain panel confirmed the high relatedness between strains of the same clade. Indeed, the genomes of both minor and core clades shared 99.9–100% ANI between members of the same clade. The tomato strain CFBP 5647 exhibited 98.5–98.7% ANI with the other strains of our panel, a value that is in the range of ANI values between each member of the core clade compared to members of the minor RNS05.1.2A clade (98.7–98.8%) (Supplementary Table S1).

The relationships between the different *D. solani* strains were further addressed by comparing the protein-coding sequences of the 17 genomes using the SiLix gene family clustering tool. Proteins were classified as homologous to others in a given family if the amino acid identities were above 70%, with 80% minimal overlap. All *D. solani* strains shared 3873 protein families representing 79% to 86% of the genome content. For the core and RNS05.1.2A clades, as many as 4216 and 4456 protein families are common, representing 91–94% and 92–95% of the genome content, respectively. Reciprocally, members of the RNS05.1.2A clade only exhibited 13 to 38 specific protein families. Members of the core clade displayed 5 to 125 specific protein families, with the highest content displayed by the strains isolated from ornamentals (25 to 125 specific protein families). For its part, the CFBP 5647 strain displayed 456 specific protein families (see below).

#### 2.2.2. Relatedness of *D. solani* Strains by SNP Analysis

**Figure 2:**
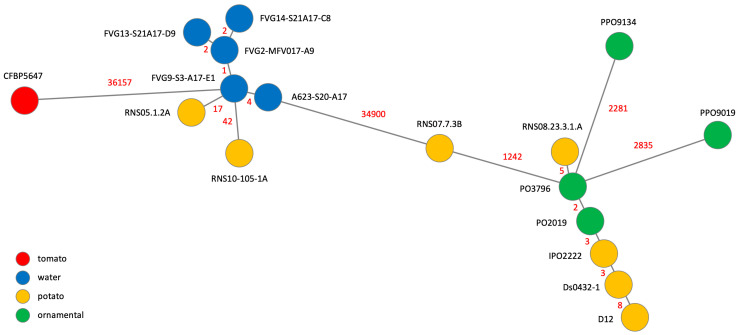
Minimum spanning tree-based genome SNP analysis. The tree is based upon 60,195 SNPs. The length of each branch (log scale) expressed in SNP numbers is indicated.

To gain further insight into *D. solani* relatedness, we constructed a Minimum Spanning Tree (MST) based on the whole set of single nucleotide polymorphisms (SNPs) present in the core genome identified in our strain panel (Figure 2).

As previously reported [12,13,14,16,17], members of the core clade only differ from each other by a few SNPs, except the potato strain RNS07.7.3B and the two ornamental strains PP09019 and PPO9134 that suffered gene replacement by horizontal gene transfer and homologous recombination with RNS05.1.2A and *D. dianthicola*, respectively. Notably, the two new genomes of strains isolated from ornamentals analysed in this study do not present such replacement and differ from the type strain IPO222 only by very few SNPs (Figure 2). Such clonality was also observed between members of the RNS05.1.2A clade. The five strains isolated from the Durance catchment only harbour 2 to 4 SNPS between each other. The two strains isolated from potato were a bit more distinct, harbouring 17 and 42 SNPs with the closest strain isolated from water.

### 2.3. Genomic Comparison of the Three D. solani Clades

#### 2.3.1. Genomic Synteny

**Figure 3:**
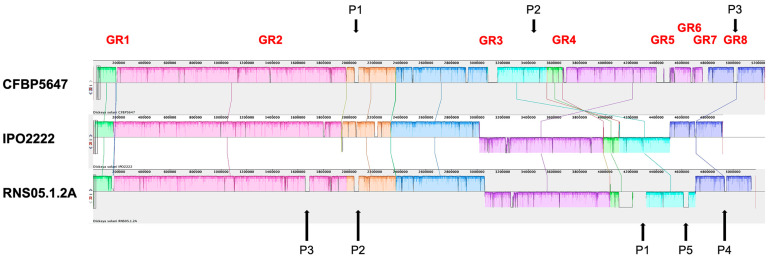
Synteny between the genomes of the three strains CFBP 5647, IPO2222 and RNS05.1.2A belonging to the three *D. solani* clades. Genomes were manually realigned to *D. solani* CFBP 5647 sequence to highlight synteny. Synteny analysis was performed using the MAUVE software v2015-02-25. P numbers indicate complete prophages as presented in Table 2. The genomic regions detected by the SiLix analysis in CFBP 5647 are indicated.

We compared the genomic architecture of the tomato strain CFBP 5647 with the type strain IPO2222 and the reference strain RNS05.1.2A using the MAUVE whole-genome alignment (Figure 3). The three genomes are highly syntenic with only one large inversion in CFBP 5647 as compared to the two other genomes. The alignment also highlighted specific regions that contain several genes related to phages and mobile genetic elements. This prompted us to predict integrated prophages based on PHASTER classification. CFBP 5647 harbours three complete prophages from which one is highly related to the Mutator phage Mu (P3, Table 2). RNS05.1.2A carries five complete prophages from which two are related to the CFBP 5647 prophage 1 (P2 and P3). RNS05.1.2A prophage P1 is included in a roughly 200,000 Kb complex repeat region (marked by a star in Figure 3) that gathers several genes linked to phages, mobile elements and methyl-accepting chemotaxix proteins (MCPs). No complete prophage was detected in IPO2222. As there were still other uncharacterised specific regions in the MAUVE alignment, we used another approach to search for clade-specific genes by using the SiLix gene family clustering tool.

#### 2.3.2. Analysis of Clade-Specific Genes

The SiLix analysis revealed that 456, 137 and 221 protein families are present only in CFBP 5647, the core clade and the RNS05.1.2A clade, respectively. More than half of the CFBP 5647-specific protein families are clustered in four genomic regions (Table 3). Two of them showed similarities with ICEs (Integrative Conjugative Element). GR3 groups 65 protein families from which ten are related to plasmid replication, recombination and segregation functions of the *Pseudomona fluorescens* mobile genomic island 01 (PFGI-1) [25]. Even if a homolog of VirD4, an ATPase involved in T-DNA transfer, is present, GR3 does not carry a Pil or T4SS-like operon necessary for the biogenesis of a pilus allowing conjugative transfer. In addition, GR3 carries mainly genes encoding hypothetical proteins and some proteins involved in metabolism. GR5 presents characteristics of a Tn4371-related ICE that is partially present in the model *D. dadantii* strain 3937 [23,26]. This ICE regroups a conjugative Trb transfer system as well as VirD2 and VirD4 associated homologs, a plasmid-replication machinery, a DNA restriction system, an efflux transport system and some proteins putatively involved in metabolism including NRPS and PKS. GR7 regroups protein families involved in a second T4SS protein secretion system and an efflux system, while GR8 regroups a complete Mu prophage. Notably, CFBP 5647 possesses a CRISPR cluster (GR6), while no such systems have been described previously in *D. solani*. All CFBP 5647 genomic regions present signs of extrachromosomal origin (Table 3).

Members of the core clade only harbour five short genomic regions (5 to 15 protein families) consisting of extrachromosomal elements, hypothetical proteins or proteins involved in metabolism. This clade is the only one whose members possess the cluster of genes encoding endotoxins of the *Bacillus thurigiensis*-related Cyt family active on insects that have been characterised in *D. dadantii* (GR3) [27]. RNS05.1.2A prophages 3 and 4 are missing in RNS10-105-1A and A623-S20-A17, respectively, and thus do not appear in clade-specific genes.

### 2.4. Virulence of New Isolates

**Figure 4:**
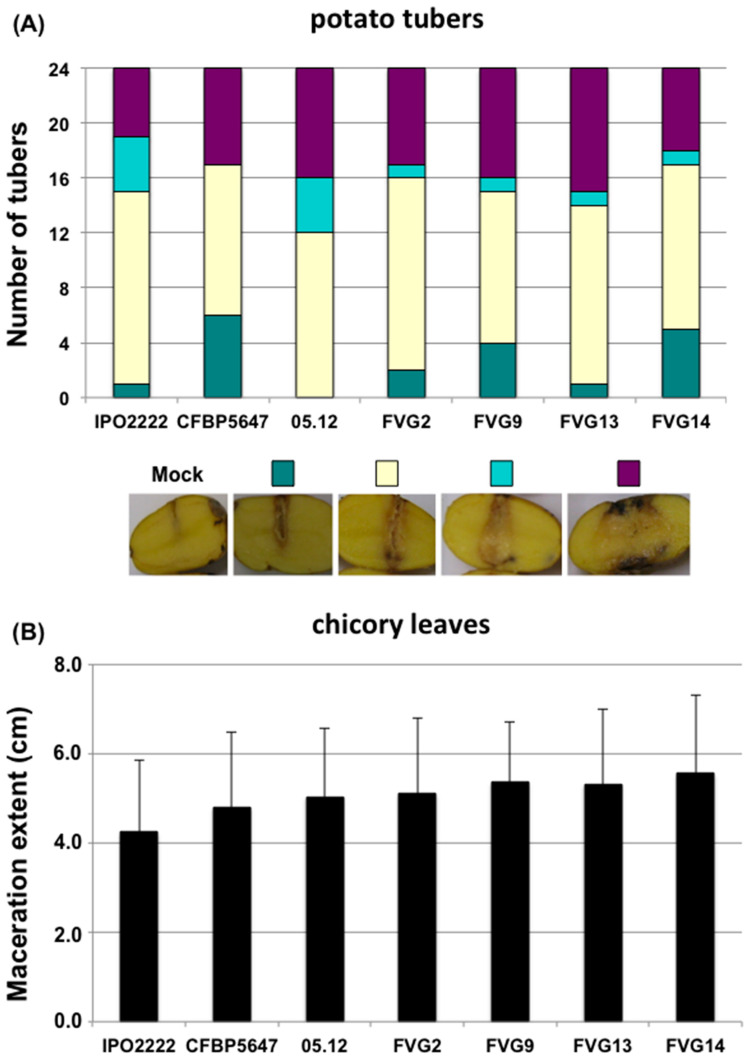
Aggressiveness of *D. solani* strains isolated from water or tomato on potato tubers and chicory leaves. For potato (**A**), symptoms were assigned to four classes according to the extent of maceration five days post-inoculation (see Material and Methods). Pictures present examples of the typology of each class. For chicory leaves (**B**), disease severity was assessed by measuring the length of macerated tissue 24 h post-inoculation. Bars: standard deviation. Both assays were performed in triplicate, and the results were pooled.

As even highly closely related *D. solani* strains might vary greatly in their aggressiveness (here defined as the quantitative production of symptoms in contrast to virulence defined as the qualitative ability of producing symptoms) [18], strains isolated from water in this study and the Caribbean tomato strain CFBP 5647 were tested for aggressiveness both on potato tubers and on detached chicory leaves (Figure 4). These strains were able to cause symptoms on both plants. Like the potato strain RNS05.1.2A, these strains were even more aggressive than the type strain IPO222 on chicory leaves (student test *p* < 0.05) (Figure 4B).

## 3. Discussion

Previous studies of *D. solani* genomic diversity [1,12,13,14,16,17] revealed the clonality of this bacterial species with the notable exception of strain RNS05.1.2A and two recently described related strains [14]. The analysed strains were, however, mainly isolated from the same host–potato–and from the same geographical area–Europe and Israel–via seed trade [28]. This points to the spread of a seminal clone that invaded the continent by seed trade dissemination. That is why in this work we extended *D. solani* genomic analysis to strains isolated from more diverse sources. The newly studied strains belong to three genomic clades exhibiting around 98% ANI values between each other: the core clade including most potato strains and two strains isolated from ornamentals, the minor clade with as reference strain RNS05.1.2A and a third “clade” defined by the strain CFBP 5647 isolated from tomato in Guadeloupe.

The two additional strains isolated from ornamentals belong to the core clade. They do not harbour signs of replacing horizontal gene transfer as described in the two previous strains analysed [16] and carry only a few SNPs as compared to other members of this clade. This reinforced the commonly accepted hypothesis that *D. solani* emerged in potato following transfer from ornamentals [29,30]. The four strains isolated from surface waters all belong to the clade exemplified by strain RNS05.1.2A. They harbour extremely few SNPs between each other and the other already analysed strain also isolated from the river Durance watershed [31]. As three of these strains have been isolated from the river Durance or canals fed by this river, we cannot exclude spreading of a single clone via the river. However, strains FVG13-S21A17-D9 and FVG14-S21A17-C8 have been isolated from a tributary of the Durance, implying at least two introduction sources in river waters. The two other members of this clade–RNS05.1.2A and RNS10-105-1A–both isolated from potato in the north of France, exhibit a few dozen SNPs with water strains and between each other. We may question if related strains are present in waterways in the north of France and might be a source of contamination explaining the occurrence of this rare clade in potato. To tackle this, it would be interesting to analyse the *D. solani* strains present in waterways in this region.

So, our study revealed both broader *D. solani* diversity with the characterisation of a third “clade” isolated in Latin America and a very high genomic conservation between clade members. In the *Dickeya* genus, diversity varies from one species to the other, with species like *D. zeae* and *D. fanghzongdai* encompassing strains so diverse that they are at the limit of species appurtenance, while other species are much more related [2]. In particular, the species *D. dianthicola*, another strain particularly studied in relation to potato outbreaks, shows both a high diversity and a high relatedness of strains isolated over a large scale of time and geographical origin [15]. As for *D. solani*, this highlights the high genomic stability for some members of this species. Similarly, in *Pectobacterium brasiliense*, Jonkheer et al. [32] reported such a high relatedness of several strains within a clade of strains isolated from different locations, besides a clade of more diverse strains. In this organism, clonality was related to a high aggressiveness. We have no indications of such a relationship in *D. solani.* Indeed, Golanovska et al. [18] showed that members of the core clade may vary greatly in their aggressiveness and, at least on detached plant organs, we did not observe differences in virulence between the three *D. solani* clades (Figure 4).

The previously reported *D. solani* core genome is very large, encompassing almost 4000 genes [1]. Adjunction of the more diverse Caribbean tomato strain CFBP 5647 did not reduce significantly this core genome, since out of the 3956 protein families present both in the core and RNS05.1.2A clades analysed in this study, only 81 are missing in CFBP 5647. As in other *Dickeya* species, the repertoire of virulence-associated genes is conserved in *D. solani* with only the presence of a split *pelN* gene in CFBP 5647.

Such a large core genome is characteristic of many *Dickeya* species in which the members share around 3500 protein families [1,2]. Notable exceptions are *D. chrysanthemi* [1], *D. oryzae* [7] and *D. dianthicola*, for which analysis of more diverse strains [15] revealed a core genome of only around 3000 protein families. This is, however, still very large if compared to, for example, *E. coli*, for which, using less stringent conditions (50% identity on 50% of the length of the proteins), the core genome was estimated to comprise only about 1500 orthologous genes [33].

Conversely, each of the three *D. solani* clades only harbouwred one to a few hundred specific genes. Many of them are clustered in genomic regions that often present signs of horizontal gene transfers or are associated with extrachromosomal elements as already reported for the core clade [12].

In conclusion, our analysis of *D. solani* showed that, besides the clonal spreading of ornamental and potato strains through Europe and Israel, another clade of clonal strains is observed, only in France until now, both in potato and in surface water. Further diversity was observed in a strain isolated from tomato in a Caribbean island. As already noticed for the related species *D. dianthicola* [15], it is thus critical in bacteria diversity studies to broaden the sources of analysed strains by varying both geographic locations and environments/hosts. The recent reports of *D. solani* occurrences in Latin America on diverse hosts [28,34,35] will clearly help to further characterise the diversity of this species.

## 4. Materials and Methods

### 4.1. dnaX-leuS-recA Phylogeny of Dickeya Solani Strains from CIRM-CFBP

Portions of genes *dnaX*, *leuS* and *recA* were sequenced following the protocol described for *Pectobacterium* isolates [36]. The sequences obtained for this study were manually checked, aligned and concatenated to reconstruct a phylogenetic tree (Figure S1). This allowed the identification of the distantly related strain CFBP 5647.

### 4.2. DNA Extraction, Genome Sequencing and Assembly

The bacterial genomes used in this study are presented in Table 1. Strains originating from water were isolated by filtering of 500 mL of surface water using 0.22 μm filters. The bacteria retained on the filters were suspended in water and serially diluted onto crystal violet pectate (CVP) medium, a semi-selective medium containing pectin that is widely used for the isolation of pectinolytic bacteria of the genera *Pectobacterium* and *Dickeya* [37]. Colonies forming pits on CVP plates were grown overnight in liquid medium (LB without NaCl), and qPCR amplifications were performed out of bacterial cell lysates with primers pelD1118d-F (VRC BTA CAA ACC SAC TCT G) and pelD1200d1-R (TGC GTT GYT RTT GAT GCT G), derived from the sequence of the gene *pelD* that is specific to the genus *Dickeya*. The *Dickeya* candidates were further purified on CVP plates and then on LB- plates (LB medium without added NaCl).

Total bacterial DNA was extracted from pure bacterial cultures using the Wizard genomic DNA purification kit (Promega) following the manufacturer’s protocol.

DNA quality checks (using the Qubit HS kit (ThermoFisher, Waltham, MA, USA) and visual quality checks on agarose gels) and genome sequencing were performed at the next-generation sequencing core facilities of the Institute for Integrative Biology of the Cell (Avenue de la Terrasse 91190 Gif-sur-Yvette France). Nextera DNA libraries were prepared from 50 ng of high-quality genomic DNA. Paired-end 2 × 75 pb sequencing was performed on an Illumina NextSeq500 apparatus with a High Output 150 cycle kit. CLC Genomics Workbench (Version 9.5.2, Qiagen Bioinformatics, Venlo, The Netherlands) was used to assemble reads. Final sequencing coverage was between 66 and 197. Genomic data for the new genome assemblies are presented in Table S2. Coding sequences were predicted and annotated using the Rapid Annotation using Subsystems Technology (RAST) server [38] with the Glimmer 3 prediction tool [39]. For the sake of comparison, genomes extracted from NCBI were imported in the RAST pipeline and re-annotated using Glimmer 3.

The complete genome of the CFBP 5647 strain was obtained using a hybrid assembly of Illumina short reads and ONT long reads. Long-read sequencing was performed on the genotoul platform (https://www.genotoul.fr; accessed 12 December 2023) with Oxford Nanopore GridION technology. The hybrid assembly of reads from the two sequencing strategies was performed using Unicycler v0.4.9 with default parameters [40].

All assemblies were deposited on the NCBI platform (https://www.ncbi.nlm.nih.gov/assembly; accessed 12 December 2023).

### 4.3. Genome Analysis

For all programs described in the following sections, default parameters were used.

Pairwise comparison of the genomes was computed using the average nucleotide identity (ANI) in the Pyani python module v0.2.10 (https://github.com/widdowquinn/pyani; accessed 12 December 2023) [41] with the BLAST algorithm (ANIb). The species threshold was set at 96%.

Orthologous sequences were clustered into homologous families using the SiLix software package v1.2.9 [42] with a 70% identity threshold and at least 80% overlap. Strain-specific and clade-specific gene families and gene families absent in only one of the analysed genomes were extracted from the SiLix output. For the construction of MLSA trees, common genes defined as genes present in all strains meeting the criteria of 70% identity threshold and at least 80% overlap were aligned using MUSCLE [43] software v5.1 and were filtered using the GBLOCK tool [44]. The alignments were used for building a phylogenetic tree with the BioNJ algorithm with SeaView software v5.0.5 [45], with 200 bootstrap replications.

### 4.4. Minimum Spanning Tree Analysis

SNPs were extracted from the aligned coding sequences of the core genome. To represent the possible evolutionary relationships between strains (minimum spanning tree), we used the online version of the software PHYLOViZ v2.0 [46].

### 4.5. MAUVE

Multiple alignments of the conserved genomic sequence were performed to analyse synteny and rearrangement events using the MAUVE software v2015-02-25 (https://darlinglab.org/mauve/mauve.html; accessed 12 December 2023) [47]. Genomes were manually realigned to the *D. solani* CFBP 5647 sequence to highlight synteny. Prophages were detected with the PHASTER search tool v4.3 [48,49].

### 4.6. Virulence Assays

Bacterial strains were plated on LB^−^ (consisting of LB medium without added NaCl (10 g/L peptone, 5 g/L yeast extract)) plates, incubated for 16 h at 28 °C and re-suspended in KPO_4_ 50 mM pH 7.0 buffer. After wounding with a yellow tip, eight potato tubers and ten detached chicory leaves for each bacterial strain were inoculated with 2 × 10^6^ bacteria and incubated at 26 °C in closed boxes to allow high humidity. After five days of incubation, symptoms on potato tubers were categorised according to the following scale: 1: maceration zone < 2 mm; 2: maceration zone < 5 mm; 3: maceration zone < 10 mm; and 4: maceration zone > 10 mm. For chicory leaves, the length of rotted tissue after 24 h incubation was measured to assess disease severity. Both assays were carried out in triplicate, and the three sets of data were pooled.

## Figures and Tables

**Table 1:** Strains used in this study.

Genomes	Host/Habitat	Country of Isolation	Year of Isolation	# Contigs	# CDS *	Accession Number
CFBP 5647	tomato	Guadeloupe, France	-	1	4883	GCA_032681045.1
PO3796	hyacinth	Netherlands	-	131	4596	GCA_033099915.1
FVG2-MFV017-A9	water	France	2017	76	4815	GCA_033100015.1
FVG9-S3-A17-E1	water	France	2017	83	4803	GCA_033099995.1
FVG13-S21A17-D9	water	France	2017	73	4829	GCA_033100035.1
FVG14-S21A17-C8	water	France	2017	77	4811	GCA_033099975.1
IPO2222^T^	potato	Netherlands	2007	1	4530	GCA_001644705.1
D12	potato	Russia	2010	30	4532	GCA_014751545.1
Ds0432-1	potato	Finland	2004	1	4498	GCA_002846975.1
RNS08.23.3.1.A	potato	France	2008	1	4536	GCA_000511285.2
RNS07.7.3B	potato	France	2007	29	4504	GCA_001401695.1
PPO9019	muscari	Netherlands	2006	2	4641	GCA_002846995.1
PPO9134	hyacinth	Netherlands	-	22	4546	GCA_001417915.1
RNS05.1.2A	potato	France	2005	1	4868	GCA_001401705.2
RNS10-105-1A	potato	France	2010	65	4712	GCA_026891515.1
A623-S20-A17	water	France	2017	3	4733	GCA_020406975.1
PO2019	hyacinth	Netherlands	2009	1	4600	GCA_017161585.1

* As defined by the RAST server (see Materials and Methods, Section 4).

**Table 2:** Complete * prophages detected in reference strains of the three *D. solani* clades.

Phage #	Length	# of Proteins	Coordinates	Closest Prophage
**CFBP 5647**				
P1	42.9 Kb	54	2038734-2081644	*Salmonella* phage SEN5
P2	62.8 Kb	59	3531751-3594647	*Staphylococcus* phage StauST398-4
P3	36.4 Kb	50	5003447-5038072	*Haemophilus* phage SuMu
**RNS05.1.2A**				
P1	27.4 Kb	35	42866-70288	Peduovirus P24B2
P2	45.9 Kb	57	2129925-2175882	*Salmonella* phage SEN5
P3	43.9 Kb	44	2520131-2564078	*Salmonella* phage SEN5
P4	38.1 Kb	52	4215256-4253442	Bacteriophage SfI
P5	47.6 Kb	71	4736805-4784434	*Edwardsiella* phage GF-2

* Prophages are considered complete if they obtained a PHASTER phage completeness score between 100 and 150.

**Table 3:** Genomic regions specific of each *D. solani* clade.

Genomic Regions	Genes ID	Coordinates	# Genes	Presence of HGT Features	Predicted Functions
**CFBP 5647** (456 specific protein families)					
GR1	181-194	178626-188439	13	yes	extrachromosomal
GR2	1260-1266	1382164-1386475	7	yes	extrachromosomal
GR3	2855-2920	3090652-3158026	65	yes	T4SS, T6SS, metabolism, PFG1-like cluster
GR4	3381-3386	3679697-3686573	6	yes	Phage-related
GR5	4075-4153	4410800-4506413	78	yes	Ice element
GR6	4313-4321	4669749-4682396	8	yes	CRISPR
GR7	4396-4445	4760648-4805394	49	yes	Efflux system, T4SS
GR8	4628-4678	5003447-5038082	50	yes	Mu prophage
**Core clade (IPO2222)** (137 specific protein families)					
GR1	519-527	594202-604799	8	no	Hypothetical
GR2	2362-2377	2609670-2622708	15	yes	Hypothetical, Phage-related
GR3	2410-2414	2655792-2658817	5	no	Cyt endotoxin
GR4	2765-2777	3011280-3023862	12	yes	Extrachromosomal
GR5	3717-3722	4059817-4065224	6	no	Metabolism, regulation
**RNS05.1.2A clade** (221 specific protein families)					
GR1	898-913	929496-951874	15	no	Hypothetical
GR2	2051-2058	2171774-2176981	7	yes	Phage-related
GR3	2237-2241	2348346-2352853	5	no	Metabolism
GR4	2258-2264	2370494-2374210	6	yes	Extrachromosomal
GR5	3791-3810	4049489-4069658	19	yes	Plasmid-related
GR6	4187-4197	4443017-4458264	10	no	Transport, hypothetical
GR7	4425-4486	4736909-4778212	61	yes	Prophage P5

## Data Availability

Data is contained within the article and supplementary material.

## Supplementary Materials

The following supporting information can be downloaded at: https://www.mdpi.com/article/10.3390/ijms242417553/s1. Reference [50] is cited in the supplementary materials.

## References

[1] Pédron J., Van Gijsegem F. (2019). Diversity in the Bacterial Genus *Dickeya* Grouping Plant Pathogens and Waterways Isolates. OBM Genet..

[2] Hugouvieux-Cotte-Pattat N., Pédron J., Van Gijsegem F. (2023). Insight into biodiversity of the recently rearranged genus *Dickeya*. Front. Plant Sci..

[3] Toth I.K., Barny M., Brurberg M.B., Condemine G., Czajkowski R., Elphinstone J.G., Helias V., Johnson S.B., Moleleki L.N., Pirhonen M., Van Gijsegem F., van der Wolf J.M., Toth I.K. (2021). Pectobacterium and *Dickeya*: Environment to Disease Development. Plant Diseases Caused by Pectobacterium and Dickeya Species.

[4] Van Gijsegem F., Hugouvieux-Cotte-Pattat N., Kraepiel Y., Lojkowska E., Moleleki L., Gorshkov V., Yedidia I., Van Gijsegem F., van der Wolf J.M., Toth I.K. (2021). Molecular interactions of *Pectobacterium* and *Dickeya* with plants. Plant Diseases Caused by Pectobacterium and Dickeya Species.

[5] Toth I.K., Barny M.-A., Czajkowski R., Elphinstone J.G., LI X.S., Pédron J., Pirhonen M., Van Gijsegem F., Van Gijsegem F., van der Wolf J.M., Toth I.K. (2021). Pectobacterium and *Dickeya*: Taxonomy and evolution. Plant Diseases Caused by Pectobacterium and Dickeya Species.

[6] Wang X., He S.W., Guo H.B., Han J.G., Thin K.K., Gao J.S., Wang Y., Zhang X.X. (2020). *Dickeya oryzae* sp. *nov*., isolated from the roots of rice. Int. J. Syst. Evol. Microbiol..

[7] Hugouvieux-Cotte-Pattat N., Van Gijsegem F. (2021). Diversity within the *Dickeya zeae* complex, identification of *Dickeya zeae* and *Dickeya oryzae* members, proposal of the novel species *Dickeya parazeae* sp. nov. Int. J. Syst. Evol. Microbiol..

[8] Alic S., Pédron J., Dreo T., Van Gijsegem F. (2019). Genomic characterization of the new *Dickeya fangzhongdai* species regrouping plant pathogens and environmental isolates. BMC Genom..

[9] Oulghazi S., Khayi S., Lafkih N., Massaoudi Y., El Karkouri A., El Hassouni M., Faure D., Moumni M. (2017). First report of *Dickeya dianthicola* causing blackleg on potato in Morocco. Plant Dis..

[10] Ge T., Jiang H., Tan E.H., Johnson S.B., Larkin R.P., Charkowski A.O., Secor G., Hao J. (2021). Pangenomic analysis of *Dickeya dianthicola* strains related to the outbreak of blackleg and soft rot of potato in USA. Plant Dis..

[11] Van der Wolf J.M., Nijhuis E.H., Kowalewska M.J., Saddler G.S., Parkinson N., Elphinstone J.G., Pritchard L., Toth I.K., Lojkowska E., Potrykus M. (2014). *Dickeya solani* Sp. *Nov*., a Pectinolytic Plant-Pathogenic Bacterium Isolated from Potato (*Solanum tuberosum*). Int. J. Syst. Evol. Microbiol..

[12] Motyka-Pomagruk A., Zoledowska S., Misztak A.E., Sledz W., Mengoni A., Lojkowska E. (2020). Comparative Genomics and Pangenome-Oriented Studies Reveal High Homogeneity of the Agronomically Relevant Enterobacterial Plant Pathogen *Dickeya solani*. BMC Genom..

[13] Blin P., Robic K., Khayi S., Cigna J., Munier E., Dewaegeneire P., Laurent A., Jaszczyszyn Y., Hong K.W., Chan K.G. (2021). Pattern and Causes of the Establishment of the Invasive Bacterial Potato Pathogen *Dickeya solani* and of the Maintenance of the Resident Pathogen *D. dianthicola*. Mol. Ecol..

[14] Khayi S., Chan K.-G., Faure D. (2022). Patterns of Genomic Variations in the Plant Pathogen *Dickeya solani*. Microorganisms.

[15] Pédron J., van der Wolf J.M., Portier P., Caullireau E., Van Gijsegem F. (2022). The broad host range plant pathogen *Dickeya dianthicola* shows a high genetic diversity. Microorganisms.

[16] Khayi S., Blin P., Pédron J., Chong T.-M., Chan K.-G., Moumni M., Hélias V., Van Gijsegem F., Faure D. (2015). Population Genomics Reveals Additive and Replacing Horizontal Gene Transfers in the Emerging Pathogen *Dickeya solani*. BMC Genom..

[17] Pédron J., Schaerer S., Kellenberger I., Van Gijsegem F. (2021). Early Emergence of *Dickeya solani* Revealed by Analysis of *Dickeya* Diversity of Potato Blackleg and Soft Rot Causing Pathogens in Switzerland. Microorganisms.

[18] Golanowska M., Potrykus M., Motyka-Pomagruk A., Kabza M., Bacci G., Galardini M., Bazzicalupo M., Makalowska I., Smalla K., Lojkowska E. (2018). Comparison of highly and weakly virulent *Dickeya solani* strains, with a view on the pangenome and panregulon of this species. Front. Microbiol..

[19] Motyka-Pomagruk A., Babinska-Wensierska W., Sledz W., Kaczorowska A.K., Lojkowska E. (2023). Phyloproteomic study by MALDI-TOF MS in view of intraspecies variation in a significant homogenous phytopathogen *Dickeya solani*. Sci. Rep..

[20] Morris C.E., Lacroix C., Chandeysson C., Guilbaud C., Monteil C., Piry S., Rochelle Newall E., Fiorini S., Van Gijsegem F., Barny M.A. (2023). Comparative abundance and diversity of populations of the *Pseudomonas syringae* and Soft Rot *Pectobacteriaceae* species complexes throughout the Durance River catchment from its French Alps sources to its delta. Peer Community J..

[21] Garlant L., Koskinen P., Rouhiainen L., Laine P., Paulin L., Auvinen P., Holm L., Pirhonen M. (2013). Genome sequence of *Dickeya solani*, a new soft rot pathogen of potato, suggests its emergence may be related to a novel combination of non-ribosomal peptide/polyketide synthetase clusters. Diversity.

[22] Brual T., Effantin G., Baltenneck J., Attaiech L., Grosbois C., Royer M., Cigna J., Faure D., Hugouvieux-Cotte-Pattat N., Gueguen E. (2023). A natural single nucleotide mutation in the small regulatory RNA ArcZ of *Dickeya solani* switches off the antimicrobial activities against yeast and bacteria. PLoS Genet..

[23] Pédron J., Mondy S., Raoul des Essarts Y., Van Gijsegem F., Faure D. (2014). Genomic and metabolic comparison with *Dickeya dadantii* 3937 reveals the emerging *Dickeya solani* potato pathogen to display distinctive metabolic activities and T5SS/T6SS-related toxin repertoire. BMC Genom..

[24] Raoul des Essarts Y., Pédron J., Blin P., Van Dijk E., Faure D., Van Gijsegem F. (2019). Common and distinctive adaptive traits expressed in *Dickeya dianthicola* and *Dickeya solani* pathogens when exploiting potato plant host. Environ. Microbiol..

[25] Mavrodi D.V., Loper J.E., Paulsen I.T., Thomashow L.S. (2009). Mobile genetic elements in the genome of the beneficial rhizobacterium *Pseudomonas fluorescens* Pf-5. BMC Microbiol..

[26] Van Houdt R., Toussaint A., Ryan M.P., Pembroke J.T., Mergeay M., Adley C.C., Roberts A.P., Mullany. P. (2011). Tn4731 ICE family of bacterial mobile genetic elements. Bacterial Integrative Mobile Genetic Elements.

[27] Costechareyre D., Balmand S., Condemine G., Rahbe Y. (2012). *Dickeya dadantii*, a plant pathogenic bacterium producing Cyt-Like entomotoxins, causes cepticemia in the pea aphid *Acyrthosiphon pisum*. PLoS ONE.

[28] Van der Wolf J.M., Acuña I., De Boer S.H., Brurberg M.B., Cahill G., Charkowski A.O., Coutinho T., Davey T., Dees M.W., Degefu Y., Van Gijsegem F., van der Wolf J.M., Toth I.K. (2021). Diseases Caused by Pectobacterium and Dickeya Species Around the World. Plant Diseases Caused by Pectobacterium and Dickeya Species.

[29] Slawiak M., van Beckhoven J.R.C.M., Speksnijde A.G.C.L., Czajkowski R.L., Grabe G., van der Wolf J.M. (2009). Biochemical and genetical analysis reveal a new clade of biovar 3 *Dickeya* spp. strains isolated from potato in Europe. Eur. J. Plant Pathol..

[30] Toth I.K., van der Wolf J.M., Saddler G., Lojkowska E., Hélias V., Pirhonen M., Tsror (Lahkim) L., Elphinstone J.G. (2011). *Dickeya* species: An emerging problem for potato production in Europe. Plant Pathol..

[31] Ben Moussa H., Bertrand C., Rochelle-Newall E., Fiorini S., Pedron J., Barny M.A. (2022). The diversity of soft rot *Pectobacteriaceae* along the Durance river stream in the south-east of France revealed by multiple seasonal surveys. Phytopathology.

[32] Jonkheer E.M., Brankovics B., Houwers I.M., van der Wolf J.M., Bonants P., Vreeburg R., Bollema R., de Haan J.R., Berke L., Smit S. (2021). The *Pectobacterium* pangenome, with a focus on *Pectobacterium brasiliense*, shows a robust core and extensive exchange of genes from a shared gene pool. BMC Genom..

[33] Lukjancenko O., Wassenaar T.M., Ussery D.W. (2010). Comparison of 61 sequenced *Escherichia coli* genomes. Microb Ecol..

[34] Leal Sanabria G., Plasencia-Márquez O., Martínez Zubiaur Y., Silvestre-Vañó M., Pérez-López E. (2023). First Report of Potato (*Solanum tuberosum*) Blackleg Disease Caused by *Dickeya solani* in Mayabeque, Cuba. Plant Dis..

[35] Rodríguez-Parra J.A., Moreno-López J.A., González-Almario A. (2022). *Dickeya solani*, *Pectobacterium atrosepticum* and *Pseudomonas asplenii*: Causal agents of bacterial soft rot in cyclamen plants (*Cyclamen persicum* Mill.) in Colombia. Can. J. Plant Pathol..

[36] Portier P., Pédron J., Taghouti G., Fischer-Le Saux M., Caullireau E., Bertrand C., Laurent A., Chawki K., Oulgazi S., Moumni M. (2019). Elevation of *Pectobacterium carotovorum* subsp. *odoriferum* to species level as *Pectobacterium odoriferum* sp. nov., proposal of *Pectobacterium brasiliense* sp. nov. and *Pectobacterium actinidiae* sp. nov., emended description of *Pectobacterium carotovorum* and description of *Pectobacterium versatile* sp. nov., isolated from streams and symptoms on diverse plants. Int. J. Syst. Evol. Microbiol..

[37] Hélias V., Hamon P., Huchet E., Wolf J.V.D., Andrivon D. (2012). Two new effective semiselective crystal violet pectate media for isolation of *Pectobacterium* and *Dickeya*. Plant Pathol..

[38] Aziz R.K., Bartels D., Best A.A., DeJongh M., Disz T., Edwards R.A., Formsma K., Gerdes S., Glass E.M., Kubal M. (2008). The RAST Server: Rapid annotations using subsystems technology. BMC Genom..

[39] Delcher A.L., Harmon D., Kasif S., White O., Salzberg S.L. (1999). Improved microbial gene identification with GLIMMER. Nucleic Acids Res..

[40] Koren S., Walenz B.P., Berlin K., Miller J.R., Phillippy A.M. (2017). Canu. Scalable and accurate long-read assembly via adaptive k-mer weighting and repeat separation. Genome Res..

[41] Pritchard L., Glover R.H., Humphris S., Elphinstone J.G., Toth I.K. (2016). Genomics and taxonomy in diagnostics for food security: Soft-rotting enterobacterial plant pathogens. Anal. Methods.

[42] Miele V., Penel S., Duret L. (2011). Ultra-fast sequence clustering from similarity networks with SiLiX. BMC Bioinform..

[43] Edgar R.C. (2004). MUSCLE: Multiple sequence alignment with high accuracy and high throughput. Nucleic Acids Res..

[44] Castresana J. (2000). Selection of Conserved Blocks from Multiple Alignments for Their Use in Phylogenetic Analysis. Mol. Biol. Evol..

[45] Gouy M., Guindon S., Gascuel O. (2010). SeaView Version 4: A Multiplatform Graphical User Interface for Sequence Alignment and Phylogenetic Tree Building. Molecular Biology and Evolution.

[46] Ribeiro-Gonçalves B., Francisco A.P., Vaz C., Ramirez M., Carriço J.A. (2016). PHYLOViZ Online: Web-based tool for visualization, phylogenetic inference, analysis and sharing of minimum spanning trees. Nucleic Acids Res..

[47] Darling A.C., Mau B., Blattner F.R., Perna N.T. (2004). Mauve: Multiple alignment of conserved genomic sequence with rearrangements. Genome Res..

[48] Arndt D., Grant J.R., Marcu A., Sajed T., Pon A., Liang Y., Wishart D.S. (2016). PHASTER: A Better, Faster Version of the PHAST Phage Search Tool. Nucleic Acids Res..

[49] Zhou Y., Liang Y., Lynch K.H., Dennis J.J., Wishart D.S. (2011). PHAST: A Fast Phage Search Tool. Nucleic Acids Res..

[50] Kumar S., Stecher G., Tamura K. (2016). MEGA7: Molecular evolutionary genetics analysis version 7.0 for bigger datasets. Mol. Biol. Evol..

