# Supplementary material for: Clonality and Diversity in the Soft Rot Dickeya solani Phytopathogen
Source: Int J Mol Sci. 2023 Dec 16;24(24):17553. doi: 10.3390/ijms242417553 (PMC10743776; doi:10.3390/ijms242417553)
Supplement: Supplementary file 1 [file ijms-24-17553-s001.zip › IJMS2023VanGijsegemetal-supplementary data/IJMS2023VanGijsegemFig.S1.docx]

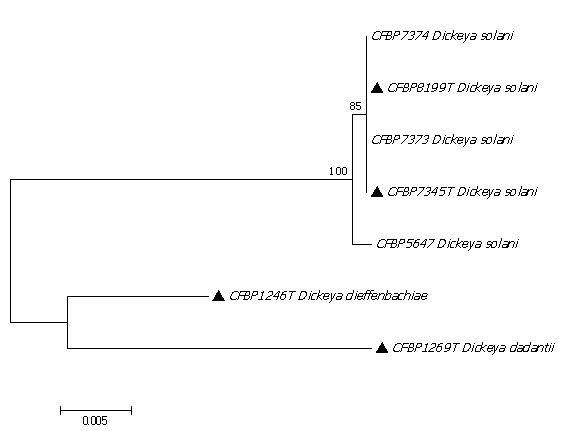


Figure S1 : **Phylogenetic tree reconstructed from partial sequences from *dnaX*, *leuS* and *recA* housekeeping genes of the *D. solani* strains held in CIRM-CFBP (https://cirm-cfbp.fr)** Sequences were obtained as described in Portier et al., 2019. Sequencing of PCR products was performed by Genoscreen (Lille, France). The consensus sequences for each gene for each strain were extracted from forward and reverse sequences assembly using Geneious Pro version 9.1.8 (www.geneious.com/). The sequences were then aligned and trimmed using BioEdit version 5.0.6. The phylogenetic tree was reconstructed with concatenated

alignments of all genes with MEGA 7.0.26 (Kumar *et al.*, 2016), using the neighbor-joining method with 1000 bootstrap replicates, and the evolutionary distances were computed by using the Kimura two-parameter method. Strain *D. dadantii* 3937 (CFBP1269) was used as a outgroup in the analysis. CFBP7345 and CFBP8199 are two occurrences of the type strain IPO2222, CFBP7345 was deposited by Jan van der Wolf and CFBP8199 by the LMG collection. CFBP7373 and CFB7374 have been isolated in Syria in 2004 from potato.

Kumar, S.; Stecher, G.; Tamura, K. MEGA7: molecular evolutionary genetics analysis version 7.0 for bigger datasets. *Mol Biol Evol* **2016**; *33*, 1870–1874.

Portier, P.; Pédron, J.; Taghouti, G.; Fischer-Le Saux, M.; Caullireau, E.; Bertrand, C.; Laurent, A.; Chawki, K.; Oulgazi, S.; Moumni, M.; et al. Elevation of *Pectobacterium carotovorum* subsp. *odoriferum* to species level as *Pectobacterium doriferum* sp. nov., proposal of *Pectobacterium brasiliense* sp. nov. and *Pectobacterium actinidiae* sp. nov., emended description of *Pectobacterium carotovorum* and description of *Pectobacterium versatile* sp. nov., isolated from streams and symptoms on diverse plants. *Int. J. Syst. Evol. Microbiol.* **2019**; *69*, 3207–3216.
